# Feasibility and outcome of metacognitive therapy for major depressive disorder: a pilot study

**DOI:** 10.1186/s12888-020-02976-4

**Published:** 2020-11-26

**Authors:** Lotta Winter, Ulrich Schweiger, Kai G. Kahl

**Affiliations:** 1 Department of Psychiatry, Social Psychiatry and Psychotherapy, Hannover Medical School, Carl-Neuberg-Str. 1, 30625 Hannover, Germany; 2 Helios Hanseklinikum Stralsund, Große Parower Str. 47-53, 18435 Stralsund, Germany

**Keywords:** Metacognitive therapy, MCT, Depression, Psychotherapy, Feasibility

## Abstract

**Background:**

Several studies have shown the effectiveness of Metacognitive Therapy (MCT) in treating different mental disorders. Most of these studies were performed in English speaking countries using the original English version of the manual. Our study aimed to examine the feasibility of the translated MCT manual in a sample of German patients with major depressive disorder.

**Methods:**

Twenty outpatients (6 male, 14 female, mean age 42.1y) with major depressive disorder were included. The main outcome was drop-out rate and satisfaction with the treatment; secondary outcomes were changes in metacognitive beliefs assessed with the metacognitive questionnaire 30 (MCQ-30), and symptom reduction measured with the Beck Depression Inventory-2 sum score (BDI-2).

**Results:**

No drop-outs during the treatment and the follow-up phase were observed. Patients and therapists were highly satisfied with MCT treatment. The MCQ-30 significantly declined over the treatment course, paralleled by a significant reduction of the BDI-2 sum scores (from 29 ± 8.6 at T0 to 8.4 ± 9.6 at the end of treatment). The average treatment duration was 10 ± 4 sessions.

**Conclusions:**

Applying the German version of the manual for Metacognitive Therapy proved to be feasible in the treatment of depressed patients in an outpatient setting. The treatment was well tolerated by German patients. Outcome in terms of reduction of depressive symptoms was good. Remarkable is the comparably short treatment duration which should be investigated further in future studies.

**Trial registration:**

German Clinical Trials Register (DRKS): DRKS00023644, 17.11.2020 (retrospectively registered).

## Background

Metacognitive Therapy (MCT) by Wells [19] describes a theory-based psychotherapy that can be applied in the treatment of several mental disorders. The treatment based on the self-regulatory model [21] aims to support the patient regain flexible attention by modulating metacognitive beliefs and reducing perservative thinking styles. In comparison to other treatments, it does not focus on content of thoughts or increasing mastery and pleasure, but on reducing unhelpful cognitive processes and facilitating metacognitive modes of processing. Results of MCT studies demonstrated considerably short treatment durations (10–12 sessions), large effect sizes and trans-diagnostic effects, as well as low drop-out rates indicating the treatment, is well tolerated, feasible and effective [4, 11]. Meanwhile, disorder specific case formulations and therapy plans have been developed for psychiatric disorders such as major depressive disorder, obsessive-compulsive disorder, generalized anxiety disorder, post-traumatic stress disorder [19] and social phobia [10], and further indications such as borderline personality disorder [9], addictive disorders [17] or psychotic disorders [15] are in development. A recent meta-analysis demonstrated superior efficacy of MCT compared to cognitive behavioral therapy (pooled effect sizes (Hedges’g) of 0.69 and 0.37 and post-treatment and follow-up), indicating that MCT might be an interesting candidate for future psychotherapeutic advances [11].

MCT is spread in Great Britain and the Scandinavian countries; however, current use in Germany is extending. Considering feasibility aspects in studies in which the original manual was used, it can be found that the treatment leads to symptom improvement and that low drop-out rates are reported [3, 8, 19]. Feasibility of MCT as delivered using the German translation of the MCT manual [20] has yet to be evaluated. Although being manualized, the flexible application of MCT strategies is essential to fit the specific patient’s needs. The exact case formulation, as well as individual combination of exercises and metaphors vary. In contrast, the first step in therapy generally is to conceptualize and socialize the patient to the maintaining processes. Profound expertise is needed to apply MCT as intended. Therefore, the aim of this study was to perform the first feasibility study on MCT in major depressive disorder in Germany. We focused particularly on drop-outs as a measure of acceptability and adherence to treatment. Secondary outcome measures were used to gather indicators for treatment effects.

## Methods

### Recruitment

**Fig. 1:**
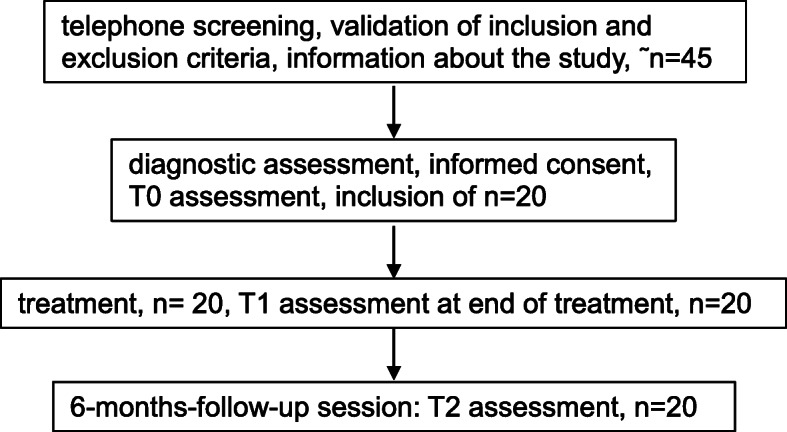
consort diagram of trial procedure

All patients were recruited from a waiting list of the psychotherapy outpatient clinic of the Department of Psychiatry, Social Psychiatry, and Psychotherapy of the Hannover Medical School. They were either referred by local psychiatrists, local general practitioners, or from other Departments of the Hannover Medical School. All patients gave written informed consent to participate in the study. As recruitment was not standardized but any patient who contacted the outpatient clinic reporting depressive symptoms was offered to take part in a screening interview, recruitment rates cannot be reported. Figure 1 shows the consort diagram of the procedure.

### Sample

Twenty patients with major depressive disorder (MDD) were included after written informed consent. MDD diagnosis was confirmed using a structured clinical interview according to DSM-IV [24], and depression severity was assessed using the German version of the Beck Depression Inventory-II [5].

Exclusion criteria were current diagnosis of a substance use disorder, psychotic disorder, bipolar disorder, any personality disorder, cognitive impairment, and acute medical conditions. To exclude patients with the aforementioned disorders, the complete structured clinical interview for DSM-IV was applied (SCID-1 and SCID-2).

Patients who were on antidepressant medication when entering the study had to be on the same doses for the past 3 months and to agree to keep the dose stable until the end of therapy.

Table 1 presents more information on sample characteristics.

**Table 1:** Baseline demographics and sample characteristics

characteristics	***N*** = 20
Age, years (SD)	42.1 (9.2)
Gender, female/male	14/6
Partnered/single	10/10
Working/ not working	18/2
Diagnosis of depression	
single depressive episode	8
recurrent major depressive disorder	12
Comorbid diagnosis	5
Panic disorder	2
General anxiety disorder	1
Obsessive-compulsive disorder	1
Post-traumatic stress disorder	1
Antidepressant medication	11
SSRI	8
SNRI	3

### MCT specific measurements

For the measurement of metacognitions, the German versions of the Metacognition questionnaire (MCQ-30, [20]) as well as the Positive Believes about Rumination Scale (PBRS, [13]) and the Negative Believes about Rumination Scale (NBRS, [14]) were administered. The MCQ-30 assesses levels of metacognitive beliefs. It has 30 items rated 1–4, with higher scores indicating higher levels of maladaptive metacognitions. Scores range from 30 to 120. The psychometric properties are adequat with Cronbach’s alpha for the total score of 0.88 [18]. The PBRS assesses beliefs about the advantages of rumination. It has nine items rated 1–4, and scores range from 9 to 36. Psychometric properties are good with a Cronbach’s alpha of 0.89 [7]. The NBRS assesses beliefs about uncontrollability and harm as well as interpersonal consequences. It has 13 items rated 1–4, and scores from 12 to 52. Good psychometric properties have been reported with Cronbach’s alpha of 0.83 [7].

### Assessment of depression severity and qualitative ratings

The severity of depressive symptoms was measured using the BDI-2. It has 21 items rated 0 to 3. The total score indicates the extent of depressive symptoms. 0 to 8 points to the prevalence of no, 9 to 13 of minimal, 14 to 18 of mild, 19 to 29 of moderate and 30 to 63 of severe depressive symptoms. Good psychometric properties have been reported with a Cronbach’s alpha of 0.93.

To receive qualitative feedback on the experience of MCT the patients and therapists were asked to reply to two qualitative questions about the therapy process by rating on a visual analogue scale at T1.

**Table:** 

Patients questions included:	
1. How satisfied were you overall with your therapy?	
2. Considering what you learned from therapy how helpful was the input to reach your personal therapy goals?	
Questions for therapists included:	
1. How satisfied were you overall with the therapy?	
2. How helpful you think was the input you gave for the patient to reach personal therapy goals?	

**Fig. 2:**
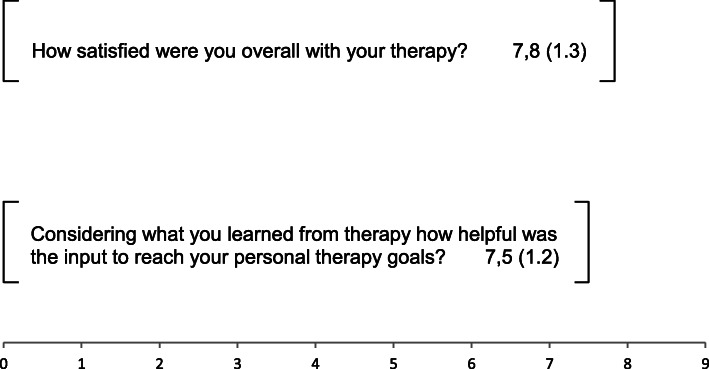
Means and standard deviation of the qualitative ratings of the patients on a visual analogue scale

**Fig. 3:**
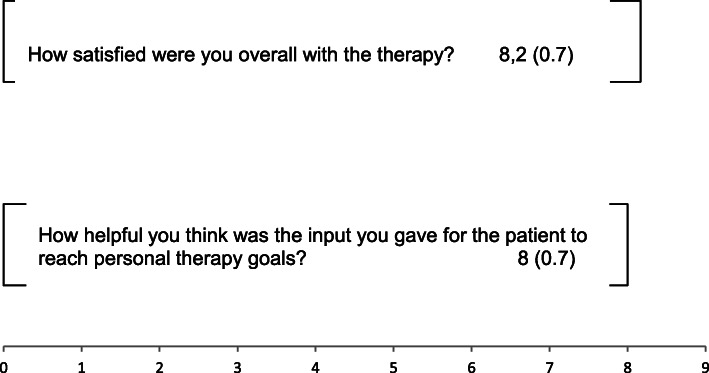
Means and standard deviations of the qualitative ratings of the therapists on a visual analogue scale

These qualitative questions could be answered with the use of a 10 cm long visual analogue scales ranging from “not at all” (0) to “very much” (9). Patients received these questions by their therapists after the last psychotherapeutic session (T1), and the psychotherapists received the qualitative questions by the study leader after the last treatment session. Results are are listed in Figs. 2 and 3.

### Assessments

All study participants were assessed before (T0) and after (T1) the treatment, as well as 6 months after therapy ended (T2). The primary outcome parameter in this pre-post-follow-up comparison was the drop-out rate as an indicator of how well the treatment was tolerated. Secondary outcomes included a change of metacognitive beliefs (MCQ-30, PBRS, NBRS) and symptom reduction (BDI-2).

### MCT treatment and therapists

All MCT therapists were graduates of the MCT Institute (www.mct-instiute.com) diploma. They were under regular supervision to ensure the quality of the delivered treatment. No formal measures of therapists’ competence, treatment integrity, or adherence were applied.

The therapy followed the translated manual and session guide of MCT in depression for individual treatment that was evaluated in our study [20]. In brief, the therapy plan consisted of conceptualization of a case formulation and socialization of the metacognitive model and mechanisms. The next step was to increase meta-awareness by identifying thoughts that act as triggers for rumination, initiating metacognitive control with the use of the attention training technique, and introducing detached mindfulness. Throughout the therapy, beliefs about the uncontrollability of rumination and worry and about threat monitoring and danger of rumination got challenged. Positive beliefs about rumination and worry were modified before relapse prevention was preceded. As the study was part of routine clinical service, therapy was terminated when individual treatment goals were reached, and the therapist and patient agreed on the end of therapy. All patients were treated in individual sessions with a duration of 50 Minutes each.

### Statistics

Data were analyzed using IBM SPSS (version 24). As the Kolmogorov-Smirnov test did not reveal normal distribution at all time points, and the sample was smaller than *n* = 30, the non-parametric Friedman test was performed to analyze differences between different time points. To define which time point differed significantly Dunn-Bonferroni test was performed as a post-hoc test. Bonferroni corrections were applied when indicated. To determine the value of the results effect sizes were calculated using Pearson’s correlations (r) which is the common way in the context of Friedman test statistics. *r* = 0.10 indicates a small, *r* = 0.30 a medium and *r* = 0.5 a large effect [1].

Descriptive analysis was performed for the whole group concerning age, gender, marital status, work status, comorbidity and visual analogue scales of the qualitative questions.

## Results

### Primary outcome

All 20 patients finished MCT after an average treatment duration of 10 sessions (SD 4.2). None of the patients dropped out, and all of them finished the T3 visit after 6 months. Only one dataset showed missing values. No adverse events were observed.

Qualitative ratings of the individual experiences with MCT in patients and therapists revealed a high extent of satisfaction, and a high valuation of the therapeutic input to reach personal therapy goals (Figs. 2 and 3).

### Secondary outcome

Table 2 presents data of all secondary outcome measures. Considering the change of metacognitive beliefs, significant reductions could be found on all three scales (MCQ-30, PBRS, NBRS) by using the Friedman-Test. Changes on the MCQ-30 and PBRS showed significant reductions in the pre to post as well as the pre to follow-up comparison. The sum score of the NBRS reduced significantly in the pre to follow-up comparison. NBRS changes in the pre to post comparison revealed a large effect size, but the reduction was not significant.

**Table 2:** Data of all secondary outcome measures

	Pre	Post	Follow-up	Friedman-Test	Dunn-Bonferroni-Tests and effect sizes T0-T1	Dunn-Bonferroni-Tests and effect sizes T0-T2
MCQ-30	61.4 ± 12.9	49.5 ± 10.8	45.5 ± 10.4	χ^2^(2) = 24.4, *p* < 0.01	z = 2.7, ***p*** **< 0.05**; *r* = 0.6	z = 4.9, ***p*** **< 0.01**, *r* = 1.1
PBRS	20.8 ± 5.5	14.4 ± 6.7	13.7 ± 7.1	χ^2^(2) = 14.4, *p* < 0.01	z = 2.7, ***p*** **< 0.05**; *r* = 0.6	z = 3.4, ***p*** **< 0.01**, *r* = 0.8
NBRS	26.9 ± 6.6	21 ± 5.6	19.3 ± 6	χ^2^(2) = 12.2, *p* < 0.01	z = 2.2, *p* = 0.09, *r* = 0.5	z = 3.4, ***p*** **< 0.01**, *r* = 0.8
BDI-2	29 ± 8.6	13 ± 8.4	8.4 ± 9.6	χ^2^(2) = 33.6, *p* < 0.01	z = 3.8, ***p*** **< 0.01**; *r* = 0.8	z = 5.7, ***p*** **< 0.01**; *r* = 1.3

*MCQ-30* Metacognitions Questionnaire-30, *PBRS* Positive beliefs about rumination scale, *NBRS* negative beliefs about rumination scale, *BDI-2* Beck Depression Inventory-2

The Friedman-Test revealed a significant reduction in BDI-2 sum-scores over time. The Dunn-Bonferroni-Tests indicate significance in the pre to post-therapy comparison as well as the pre to follow-up comparison. Effect sizes for both comparisons are large.

**Fig. 4:**
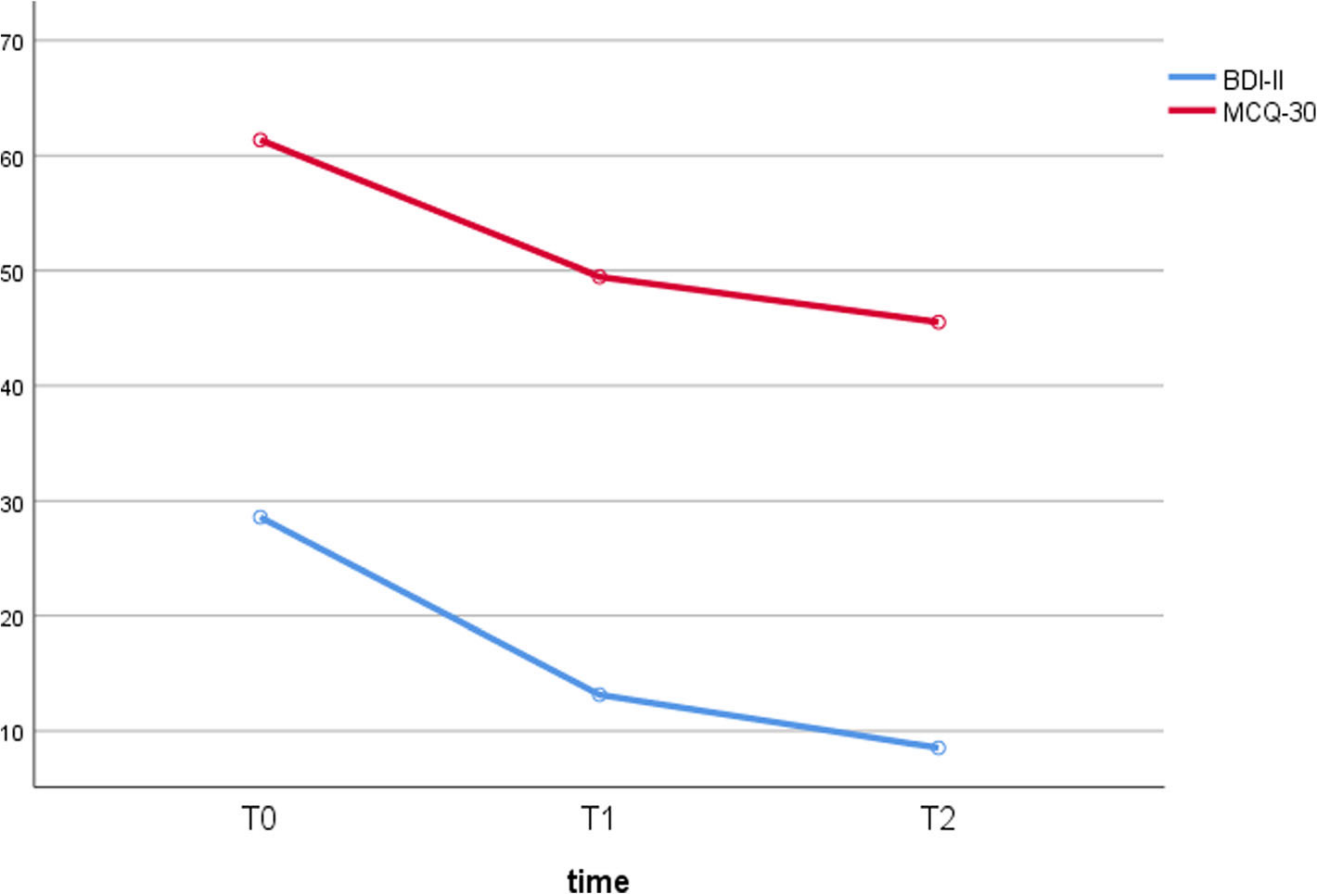
courses of BDI-2 scores and MCQ-30 scores

Figure 4 shows that courses of BDI-2 scores and MCQ-30 scores are similar.

## Discussion

We here demonstrate the feasibility of the German version of the MCT manual in patients with major depressive disorder. None of the patients dropped out or requested a change of therapy, and patients were highly satisfied with MCT treatment. These findings are in line with feasibility studies on the original manual [3, 8, 19].

Results from the secondary outcomes provide preliminary indications of efficacy of MCT in depressive patients, demonstrated by a significant reduction in BDI-2 scores after the end of treatment. In line with the assumption that changes in metacognitive beliefs underlie the antidepressive effect of MCT, the MCQ-30 sum score declined significantly (Fig. 1). Concerning lasting effects of MCT treatment, BDI-2 sum scores further declined at last follow-up 6 months after the end of treatment, Further, all patients who were classified as remitters (≥50% symptom reduction in BDI-2 sum score) at the end of treatment were still in remission at follow-up after 6 months.

Our results are in accordance with others demonstrating the clinical efficacy of MCT [2, 6, 16, 22]. A recent meta-analysis integrating data of 15 trials demonstrated large effect sizes of MCT against waitlist condition (Hedges’ *g* = 2.06), and even superior pooled effect sizes compared to cognitive and behavioral interventions at post-treatment and at follow-up (Hedges’ g 0.69 and 0.37 respectively) [11].

Given the high level of health care expenditure for patients with major depressive disorder, our findings suggest a potential time effectiveness of this therapeutic intervention. For the treatment of depression, the manual suggests 8 to 10 sessions. In our trial, average treatment duration was ten sessions. Compared to other psychotherapies, such as cognitive-behavioral therapy (CBT) or psychodynamic therapy, treatment duration seems shorter in MCT. A recent study demonstrated that in general the average treatment duration of CBT is 40 sessions and of psychodynamic therapy is 62 sessions [12]. One possible explanation for the comparably small number of sessions needed in MCT is that underlying processes are modified rather than content or schema-related aspects of a disorder [23].

Limitations: The lack of a control group, the limited number of patients and the individual medication histories hampered the interpretation of the data. In addition there might be a selection bias as we can not rule out that patients with benevolent attitudes concerning psychotherapy were mainly included. The observation period was relatively short due to the feasability study design. Further studies should include long-term observations to rule out short-term positive effects of MCT treatment. Pharmacotherapy – although guideline-based – could not be standardized during the study and might have affected treatment outcome. However, more studies with greater cohorts of patients, directly comparing different psychotherapeutic methods are warranted. In addition, future studies should evaluate the quality criteria of the translated questionnaires and use formal measures of therapists’ competence and adherence.

## Conclusions

Applying the German version of the manual for Metacognitive Therapy is feasible in the treatment of depressed patients in an outpatient setting. The treatment was well tolerated by German patients and accounted as helpful by the therapists. Outcome in terms of reduction of metacognitive beliefs and depressive symptoms was good. Remarkable is the comparably short treatment duration which should be investigated further in future studies.

## Data Availability

The datasets used and analysed during the current study are available from the corresponding author on reasonable request.

## Abbreviations

MCT: Metacognitive Therapy
MCQ-30: Metacognitions questionnaire 30
BDI-2: Beck Depression Iventory-2
MDD: Major depressive disorder
SCID-1: Structural clinical interview for DSM-IV
SCID-2: Structural clinical interview for DSM-IV
PBRS: Positive beliefs about rumination scale
NBRS: Negative beliefs about rumination scale
CBT: Cognitive behavioral therapy
VAS: Visual analogue scale

## References

[1] Cohen J. A power primer. Quant Meth Psychol. 1992;112:155–9. 10.1037//0033-2909.112.1.155 19565683

[2] Dammen T, Papageorgiou C, Wells A (2016). A Two Year Follow up Study of Group Metacognitive Therapy for Depression in Norway. J Depress Anxiety.

[3] Fisher PL, Wells A (2008). Metacognitive therapy for obsessive-compulsive disorder: a case series. J Behav Ther Exp Psychiatry.

[4] Hagen R, Hjemdal O, Solem S, Kennair LEO, Nordahl HM, Fisher P (2017). Metacognitive Therapy for Depression in Adults: A Waiting List Randomized Controlled Trial with Six Months Follow-Up. Front Psychol.

[5] Hautzinger M, Keller F, Kuehner C (2006). Beck-depressions-Inventar: revision.

[6] Hjemdal O, Solem S, Hagen R, Kennair LEO, Nordahl HM, Wells A (2019). A randomized controlled trial of metacognitive therapy for depression: analysis of 1-year follow-Up. Front Psychol.

[7] Luminet O, Papageorgiou C, Wells A (2004). Assessment and measurement of rumination. Rumination: Nature, Theory, and Treatment of Negative Thinking in Depression.

[8] Morrison AP, Pyle M, Chapman N, French P, Parker SK, Wells A (2014). Metacognitive therapy in people with a schizophrenia spectrum diagnosis and medication resistant symptoms: a feasibility study. J Behav Ther Exp Psychiatry.

[9] Nordahl HM. The ERIS protocol. MCT for Borderline Personality. Manchester: Paper presented at the MCT conference; 2011.

[10] Nordahl H, Wells A (2017). Testing the metacognitive model against the benchmark CBT model of social anxiety disorder: Is it time to move beyond cognition. PLoS One.

[11] Normann N, Morina N (2018). The efficacy of metacognitive therapy: a systematic review and meta-Analysis. Front Psychol.

[12] Nübling, R., Jeschke, K., Ochs, M., and Schmidt, J. (2014). Zur ambulanten psychotherapeutischen Versorgung in Deutschland. Eine Befragung von Psychotherapeutinnen und Psychotherapeuten in fünf Bundesländern als ein Beitrag zur psychotherapeutischen Versorgungsforschung 2014, DPTVDPTV.

[13] Papageorgiou C, Wells A (2001). Positive beliefs about depressive rumination: development and preliminary validation of a self-report scale. Behav Ther.

[14] Papageorgiou C, Wells A (2001). Metacognitive beliefs about rumination in recurrent major depression. Cogn Behav Pract.

[15] Parker SK, Mulligan LD, Milner P, Bowe S, Palmier-Claus JE (2020). Metacognitive therapy for individuals at high risk of developing psychosis: a pilot study. Front Psychol.

[16] Solem S, Kennair LEO, Hagen R, Havnen A, Nordahl HM, Wells A (2019). Metacognitive therapy for depression: a 3-year follow-up study assessing recovery, relapse, work force participation, and quality of Life. Front Psychol.

[17] Spada MM, Wells A (2009). A metacognitive model of problem drinking. Clin Psychol Psychother.

[18] Spada MM, Mohiyeddini C, Wells A (2008). Measuring metacognitions associated with emotional distress: factor structure and predictive validity of the metacognitions questionnaire 30. Personal Individ Differ.

[19] Wells A (2009). Metacognitive therapy for anxiety and depression.

[20] Wells A (2011). Metakognitive Therapie bei Angststörungen und Depression.

[21] Wells A, Matthews G (1994). Attention and emotion: a clinical perspective.

[22] Wells A, Fisher P, Myers S, Wheatley J, Patel T, Brewin CR (2012). Metacognitive therapy in treatment-resistant depression: a platform trial. Behav Res Ther.

[23] Winter L, Gottschalk J, Nielsen J, Wells A, Schweiger U, Kahl KG (2019). A comparison of metacognitive therapy in current versus persistent depressive disorder - a pilot outpatient Study. Front Psychol.

[24] Wittchen H, Zaudig M, Fydrich T (1997). SKID-I und SKID-II. Strukturiertes Klinisches Interview für DSM-IV.

